# Correction: No increase in use of hospitals for childbirth in Tanzania over 25 years: Accumulation of inequity among poor, rural, high parity women

**DOI:** 10.1371/journal.pgph.0001976

**Published:** 2023-05-17

**Authors:** Manuela Straneo, Lenka Beňová, Thomas van den Akker, Andrea B. Pembe, Tom Smekens, Claudia Hanson

In Fig 1, a second panel was omitted. See a corrected Fig 1 here.

**Fig 1:**
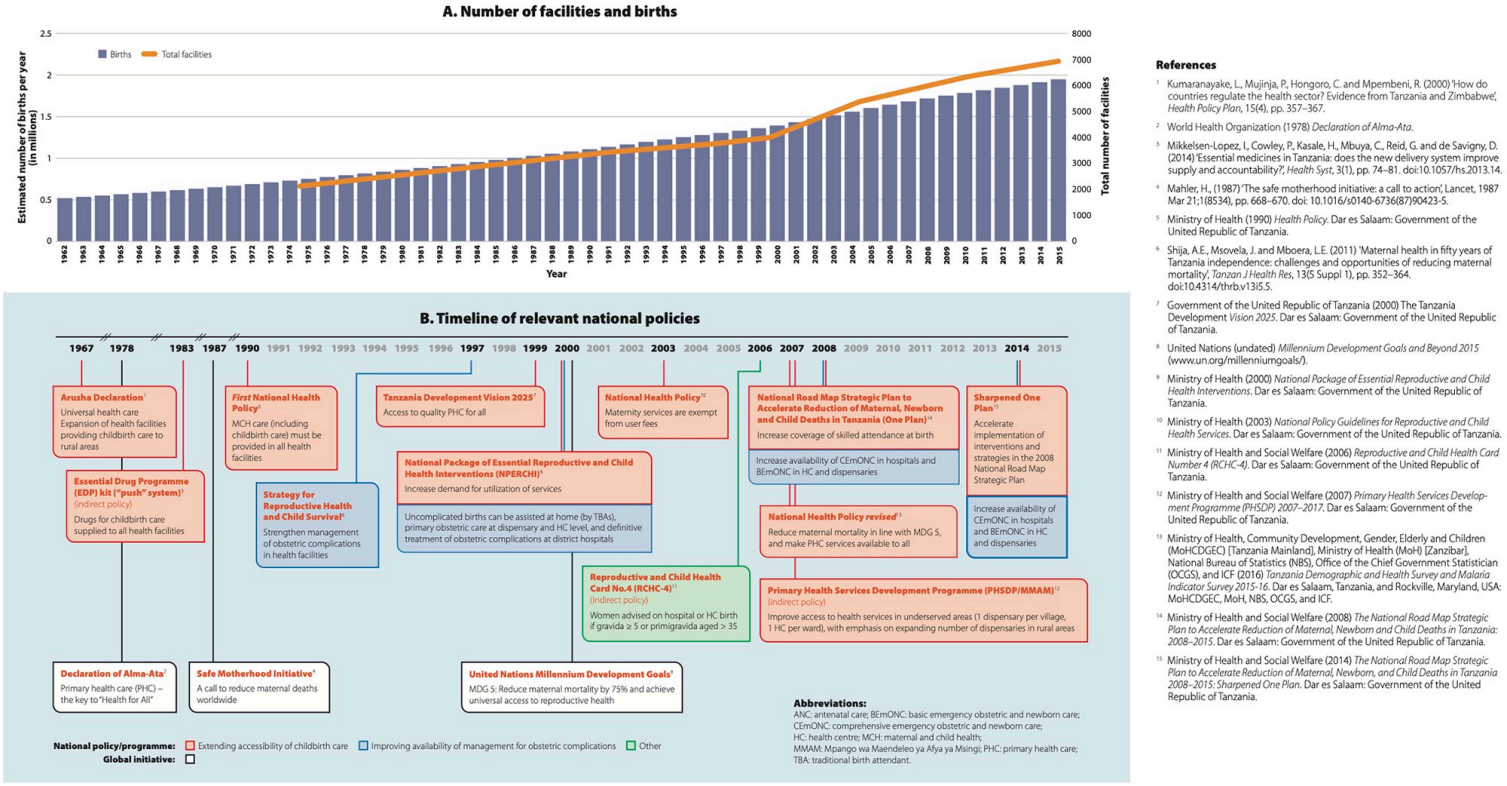
Trend of population and health facility numbers since independence in Tanzania (A) and timeline of relevant policies (B).
