# Supplementary material for: No increase in use of hospitals for childbirth in Tanzania over 25 years: Accumulation of inequity among poor, rural, high parity women
Source: PLOS Glob Public Health. 2022 Aug 15;2(8):e0000345. doi: 10.1371/journal.pgph.0000345 (PMC10021586; doi:10.1371/journal.pgph.0000345)
Supplement: Attachment — Submitted filename: Response to reviewers.docx [file pgph.0000345.s006.docx]

To Shailendra Prasad, MD, MPH
Academic Editor
PLOS Global Public Health

PGPH-D-22-00216
No increase in use of hospitals for childbirth in Tanzania over 25 years: accumulation of inequity among poor, rural, high parity women
PLOS Global Public Health


Dear Dr Prasad

Thank you for your letter regarding our manuscript. We are happy to hear you felt it has merit.

We wish to thank you and the reviewers for the time taken to read through it. We appreciate the comments made and have amended the manuscript to address the points raised. The manuscript has been improved by the reviews.

Please find below our responses:

Editor/reviewers comments *in italics*

Our response **in bold**

Modified text in blue

*Thank you for submitting your manuscript to PLOS Global Public Health. After careful consideration, we feel that it has merit but does not fully meet PLOS Global Public Health’s publication criteria as it currently stands. Therefore, we invite you to submit a revised version of the manuscript that addresses the points raised during the review process.

My compliments on a well written manuscript. One of our reviewers had no suggested edits.*

**Thank you very much!** *Please see below for the edits suggested by the other reviewer -*

"*Under methods and lines 131-135 need revising because line 133 refers to "the third and last survey which were referred to as 2004 and 2015..." This could be rephrased because it is not clear what the word "last" refers to*”.

Reply: **thank you for this comment, which helps to clarify the methods. It has been modified, and the relevant paragraph is pasted below, with changes in blue**.

Amended text: “We used Demographic and Health Surveys (DHS), nationally representative, household-based cross-sectional surveys, which provide socio-demographic and health utilisation data mainly for women and children. We included all Tanzania DHS surveys with a household wealth variable: 1996, 1999, 2004-5, 2010, 2015-16 (6, 22-25). The period analysed spans 1991-2016 (25 years), as DHS include data from women whose most recent birth was within 5 years of the survey. The 2004-5 survey is referred to as 2004 and the most recent survey is referred to as 2015, which correspond to the years when most data were collected.”

*Under ethical approval line 197, there seems a need to specify which ethics committees, boards or official titles granted permissions for the study. Ethics approval by "DHS program", line 200, appears too broad in this context and may be revised.*

Reply: **thank you for raising this point. The ethical approval section has been reworded, and now reads:**

Amended text: The Demographic and Health Survey data collection procedures were reviewed and approved by the ICF Institutional Review Board (30). The surveys are conducted by the Tanzanian government (National Bureau of Statistics or Ministry of Health, Community Development, Elderly, Gender and Children in 2015-16) and participants provided informed consent and assurance of confidentiality before the interviews (30). Permission to study the datasets for this research was obtained in writing from the DHS program. The anonymised dataset was downloaded and used for this study.

*Results, line 202, are derived from already collected data from surveys covering the reported 25 years as indicated. It is assumed that the data was raw in that it was never analysed and interpreted to address the current research question. in that sense, this is viewed as primary scientific research.*

Reply: **yes, this is indeed true. The term secondary has been removed from line 199. The sentence now reads**:

Amended text: “Permission to study the datasets for this research was obtained in writing by the DHS program, and all datasets downloaded were anonymized”

*Under line 382 strengths and limitations, inequity and the vulnerable population at risk was addressed adequately. Nevertheless, the preference for the current statistical tool may need to comment on as SPSS appears superior in social and medical sciences."

I recommend that you address the first three issues that were brought up by the reviewer. I understand the thoughts behind the last recommendation, please do not feel obliged to address it.*

Reply: **thank you for this comment.**

**In addition to the changes outlined above, minor edits were made to improve clarity (they are marked in the track changes manuscript).**

Please submit your revised manuscript by . If you will need more time than this to complete your revisions, please reply to this message or contact the journal office at globalpubhealth@plos.org. When you're ready to submit your revision, log on to https://www.editorialmanager.com/pgph/ and select the 'Submissions Needing Revision' folder to locate your manuscript file.

Please include the following items when submitting your revised manuscript:

- A rebuttal letter that responds to each point raised by the editor and reviewer(s). You should upload this letter as a separate file labeled 'Response to Reviewers'.
- A marked-up copy of your manuscript that highlights changes made to the original version. You should upload this as a separate file labeled 'Revised Manuscript with Track Changes'.
- An unmarked version of your revised paper without tracked changes. You should upload this as a separate file labeled 'Manuscript'.

Guidelines for resubmitting your figure files are available below the reviewer comments at the end of this letter.

We look forward to receiving your revised manuscript.

Kind regards,

Shailendra Prasad, MD, MPH
Academic Editor
PLOS Global Public Health

Journal Requirements:

*1. Please amend your detailed Financial Disclosure statement. This is published with the article, therefore should be completed in full sentences and contain the exact wording you wish to be published.*

*If you did not receive any funding for this study, please simply state: “The authors received no specific funding for this work.”*

**The sentence “The authors received no specific funding for this work.” has been added.**

*2. Please update the 'Competing Interests' statement with this "The authors have declared that no competing interests exist".*

**The sentence "The authors have declared that no competing interests exist" has been added.**

*3. In the online submission form, you indicated that “Secondary analysis of publicly available data”. All PLOS journals now require all data underlying the findings described in their manuscript to be freely available to other researchers, either 1. In a public repository, 2. Within the manuscript itself, or 3. Uploaded as supplementary information.*

**This has been amended as follows:**

The DHS dataset is freely available for use upon request at <https://dhsprogram.com/data/available-datasets.cfm>. After the request has been approved, a de-identified dataset is made available. The authors confirm they had no special access or privileges that others would not have.

*This policy applies to all data except where public deposition would breach compliance with the protocol approved by your research ethics board. If your data cannot be made publicly available for ethical or legal reasons (e.g., public availability would compromise patient privacy), please explain your reasons by return email and your exemption request will be escalated to the editor for approval. Your exemption request will be handled independently and will not hold up the peer review process, but will need to be resolved should your manuscript be accepted for publication. One of the Editorial team will then be in touch if there are any issues.*

*Please review your reference list to ensure that it is complete and correct. If you have cited papers that have been retracted, please include the rationale for doing so in the manuscript text, or remove these references and replace them with relevant current references. Any changes to the reference list should be mentioned in the rebuttal letter that accompanies your revised manuscript. If you need to cite a retracted article, indicate the article’s retracted status in the References list and also include a citation and full reference for the retraction notice.*

**The references have been revised and, where necessary, edited. The following changes were made**

**Reference 1: author institutions not reported correctly. It now reads:**

WHO, UNICEF, UNFPA, World Bank Group, United Nations Population Division. Trends in maternal mortality: 1990 to 2015: estimates by WHO, UNICEF, UNFPA, World Bank Group and the United Nations Population Division. Geneva: World Health Organization; 2015.

**Reference 6: author institutions were not reported correctly: It has been correct, and now reads:**

Ministry of Health, Community Development, Gender, Elderly and Children - MoHCDGEC/Tanzania Mainland, Ministry of Health - MoH/Zanzibar, National Bureau of Statistics - NBS/Tanzania, Office of Chief Government Statistician - OCGS/Zanzibar, ICF. Tanzania Demographic and Health Survey and Malaria Indicator Survey 2015-2016. Dar es Salaam, Tanzania: MoHCDGEC, MoH, NBS, OCGS, and ICF; 2016.

**Reference 14: author institution was reported as an abbreviation. It has been edited, and now reads:**

Ministry of Health. HEALTH POLICY. Dar es Salaam: United Republic of Tanzania; 1990.

**Reference 15: author institution was reported as an abbreviation and the date of publication was not reported correctly. It now reads:**

Ministry of Health and Social Welfare. PRIMARY HEALTH SERVICES DEVELOPMENT PROGRAMME. Dar es Salaam: The United Republic of Tanzania; 2007

**Reference 20: author institution was reported as an abbreviation. It has been edited, and now reads:**

Ministry of Health and Social Welfare. Reproductive and child health card number 4 (RCHC-4). Dar es Salaam: Ministry of Health and Social Welfare, The United Republic of Tanzania 2006.

**Reference 29: volume and pages were missing, and have been added.**

Straneo M, Benova L, Hanson C, Fogliati P, Pembe AB, Smekens T, et al. Inequity in uptake of hospital-based childbirth care in rural Tanzania: analysis of the 2015-16 Tanzania Demographic and Health Survey. Health Policy Plan. 2021;36:1428-1440

**Reference 33 was inserted:**

DHS program. Protecting the Privacy of DHS Survey Respondents [June 26, 2022] Available from: <https://dhsprogram.com/Methodology/Protecting-the-Privacy-of-DHS-Survey-Respondents.cfm>

**Reference 50 was missing volume & page number. It has been corrected, and now reads:**

50.	Roder-DeWan S. Decentralization and Regionalization: Redesigning Health Systems for High Quality Maternity Care Comment on "Decentralization and Regionalization of Surgical Care: A Review of Evidence for the Optimal Distribution of Surgical Services in Low- and Middle-Income Countries". Int J Health Policy Manag. 2020;10(4):215-217.

**Reference 52 was edited, and now reads:**

Gilson L, Doherty J, Loewenson R, Francis V. Challenging inequities through health systems. Final report. Knowledge Network on Health Systems. WHO Commission on the Social Determinants of Health; 2007

**Comments to the Author**

1. Does this manuscript meet PLOS Global Public Health’s [publication criteria](https://journals.plos.org/globalpublichealth/s/criteria-for-publication)? Is the manuscript technically sound, and do the data support the conclusions? The manuscript must describe methodologically and ethically rigorous research with conclusions that are appropriately drawn based on the data presented.

Reviewer #1: Yes

Reviewer #2: Yes

2. Has the statistical analysis been performed appropriately and rigorously?

Reviewer #1: Yes

Reviewer #2: Yes

3. Have the authors made all data underlying the findings in their manuscript fully available (please refer to the Data Availability Statement at the start of the manuscript PDF file)?

The [PLOS Data policy](https://journals.plos.org/globalpublichealth/s/data-availability) requires authors to make all data underlying the findings described in their manuscript fully available without restriction, with rare exception. The data should be provided as part of the manuscript or its supporting information, or deposited to a public repository. For example, in addition to summary statistics, the data points behind means, medians and variance measures should be available. If there are restrictions on publicly sharing data—e.g. participant privacy or use of data from a third party—those must be specified.

Reviewer #1: Yes

Reviewer #2: Yes

4. Is the manuscript presented in an intelligible fashion and written in standard English?

PLOS Global Public Health does not copyedit accepted manuscripts, so the language in submitted articles must be clear, correct, and unambiguous. Any typographical or grammatical errors should be corrected at revision, so please note any specific errors here.

Reviewer #1: Yes

Reviewer #2: Yes

5. Review Comments to the Author

**please see above**

6. PLOS authors have the option to publish the peer review history of their article ([what does this mean?](https://journals.plos.org/globalpublichealth/s/editorial-and-peer-review-process#loc-peer-review-history)). If published, this will include your full peer review and any attached files.


**Do you want your identity to be public for this peer review?** If you choose “no”, your identity will remain anonymous but your review may still be made public.


For information about this choice, including consent withdrawal, please see our [Privacy Policy](https://www.plos.org/privacy-policy).

Reviewer #1: **Yes:** John Mukuka Musonda

Reviewer #2: No
